# Research progress on the relationship between traumatic brain injury and brain‐gut‐microbial axis

**DOI:** 10.1002/ibra.12153

**Published:** 2024-03-30

**Authors:** Jie Yu, Yun‐Xin Chen, Jin‐Wei Wang, Hai‐Tao Wu

**Affiliations:** ^1^ Department of Neurosurgery Affiliated Hospital of Zunyi Medical University Zunyi Guizhou China

**Keywords:** brain‐gut‐microbial axis, fecal microbiota transplantations, microbes, nervous system, traumatic brain injury

## Abstract

Traumatic brain injury (TBI) is a common disease with a high rate of death and disability, which poses a serious threat to human health; thus, the effective treatment of TBI has been a high priority. The brain‐gut‐microbial (BGM) axis, as a bidirectional communication network for information exchange between the brain and gut, plays a crucial role in neurological diseases. This article comprehensively explores the interrelationship between the BGM axis and TBI, including its physiological effects, basic pathophysiology, and potential therapeutic strategies. It highlights how the bidirectional regulatory pathways of the BGM axis could provide new insights into clinical TBI treatment and underscores the necessity for advanced research and development of innovative clinical treatments for TBI.

## Graphical abstract

Here, we review the relationship between the brain and the gut following craniocerebral trauma, and we examine the brain‐gut‐microbial axis pathway. Our aim is to propose potential treatments for traumatic brain injury (TBI), including microbial intervention, microbiota transplantation, vagus nerve stimulation, and so forth, with the hope that they may be applied in clinical practice in the future to provide greater benefits to TBI patients.

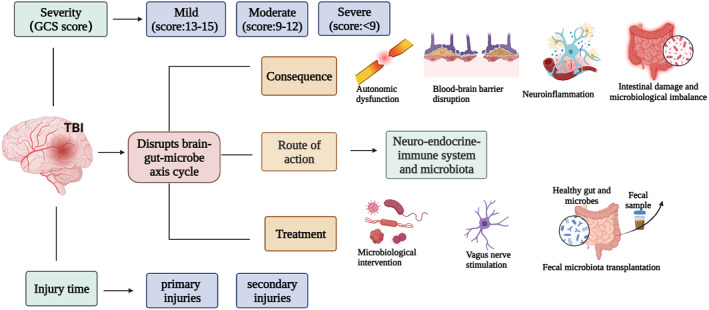

## 1 INTRODUCTION

Traumatic brain injury (TBI) is a significant contributor to global mortality and morbidity. Annually, over 50 million people worldwide are affected by brain trauma, with 10 million of them dying and almost 30 million suffering from long‐term physical disabilities.^1^, ^2^ The social and economic burden of TBI has placed it at the forefront of public attention, but decades of costly research have yielded only limited clinical results due to a poor understanding of TBI's heterogeneity and complexity.

Physiological effects following TBI have been increasingly studied, with intestinal dysfunction being recognized as an important manifestation.^3^ Furthermore, secondary gastrointestinal dysfunction may exacerbate mortality in TBI.^4^ The brain‐gut‐microbial (BGM) axis plays a key role in these physiological responses as a bridge for information exchange between the brain and the gut. It incorporates both afferent and efferent signals, involving neuronal, hormonal, and immunologic pathways.^5^ Initiating enteral nutrition early in patients with TBI not only leads to improved neurological outcomes but also reduces the risk of multiorgan dysfunction and infection when compared to delayed enteral or parenteral nutrition.^6^ Probiotics and prebiotics can reduce the inflammatory response in the brain following a TBI.^7^ The studies mentioned above increasingly suggest that the gastrointestinal tract is a novel avenue for enhancing neurological function and prognosis in TBI. More attention is being devoted to the close relationship between the BGM axis and TBI. This article reviews the various regulatory pathways of the BGM axis in TBI and summarizes potential treatments based on this foundation. The goal is to provide new insights into the clinical treatment of TBI.

## 2 BRIEF OVERVIEW AND PATHOPHYSIOLOGY OF TBI

TBI refers to the structural and physiological disruption of normal brain functioning due to external forces. The primary causes of TBI include falls, traffic accidents, sports‐related injuries, and explosive blasts encountered in combat zones. As a silent epidemic, TBI is the leading cause of death in individuals under the age of 45 in the Western World and is a contributing factor in 30.5% of all injury‐related deaths in the United States.^8^ Direct medical expenditures and indirect costs attributable to TBI reached upwards of $60 billion in the year 2000, creating a significant burden on both the community and the economy.^9^ TBI severity is classified based on the Glasgow Coma Scale, where patients are assessed using clinical symptoms. Their overall score is then calculated, classifying their injury as either mild (score: 13–15), moderate (score: 9–12), or severe (score: <9). Patients can experience behavioral and cognitive dysfunction, which can significantly impact their health and quality of life, even in cases of mild TBI.

TBI can be classified into primary and secondary injuries. Primary injuries result from direct mechanical damage to the brain tissue caused by external forces, such as cerebral contusion and cerebral hemorrhage. Secondary injuries occur within minutes of the primary injury and can persist for days, months, or even years.^10^ These injuries typically lead to neuroinflammation, neurodegeneration, and neurological dysfunctions.^11^ The process involves the disruption of intracellular ions (e.g., Ca^2+^, Na^+^, K^+^) and the release of excitotoxic neurotransmitters (e.g., glutamate), as well as the breakdown of the blood–brain barrier (BBB).^12^ This process leads to cerebral edema, oxidative stress, and neuroinflammation. Upon disruption of the BBB, peripheral immune cells such as leukocytes could enter the brain parenchyma. They may eventually activate resident glial cells such as microglia and astrocytes, leading to neuroinflammation and potentially exacerbating brain damage.^13^ However, the relations between the brain and gut following injuries remain to be discussed.

## 3 AN OVERVIEW OF THE BGM AXIS

Actually, the brain and the gut form an intimate connection as early as embryonic development, because both the brain and the gut originate from the neural crest of the early embryo. As the embryo develops, a portion of it forms the central nervous system (CNS), while the other part becomes the enteric nervous system (ENS).^14^ The ENS contains hundreds of millions of nerve cells and is often referred to as the “second brain” of the human body.^15^ In 1910, George Porter Phillips discovered that taking live, beneficial bacteria (probiotics) helped in treating depression, unveiling the BGM axis doctrine.

**Figure 1:**
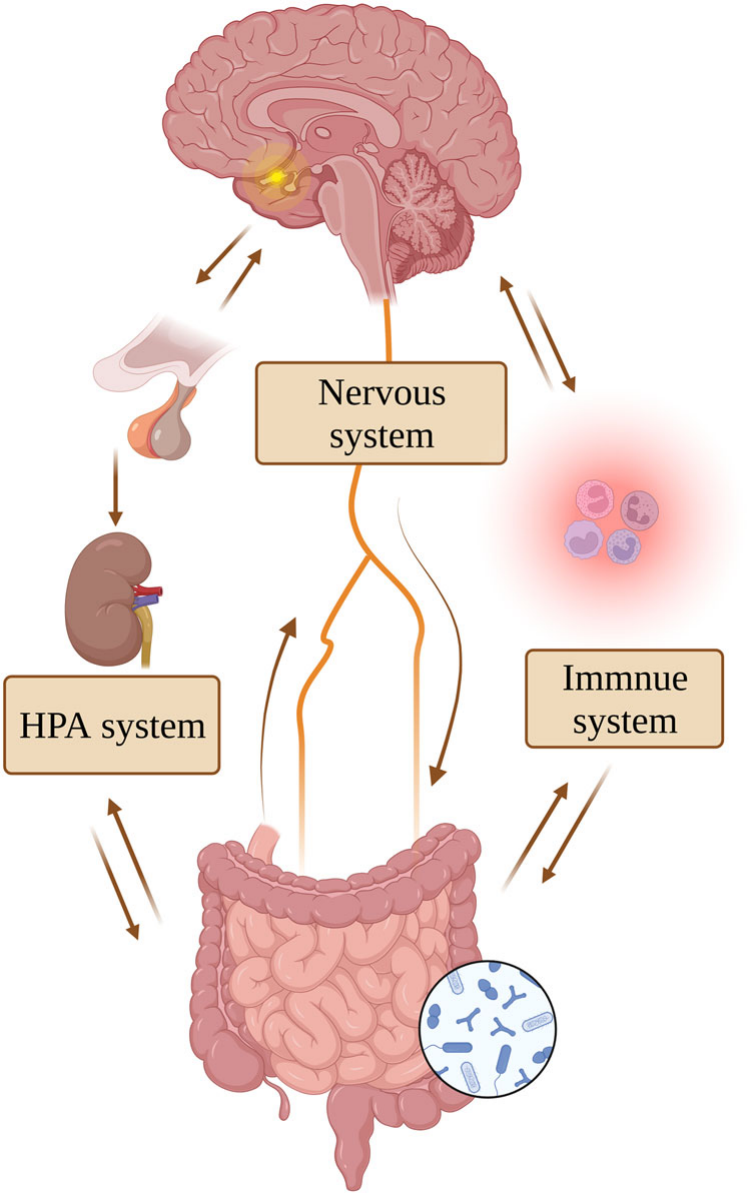
Brain‐gut‐microbial axis circulation. Bidirectional regulation between the brain and the gut primarily through the nervous system, the endocrine system, and the immune system. HPA, hypothalamic–pituitary–adrenal. [Color figure can be viewed at wileyonlinelibrary.com]

The BGM axis consists of many signaling molecules, gastrointestinal mucosal cells, autonomic nerves, and the BBB. It is critical for CNS and gastrointestinal homeostasis and regulates a wide range of functions, including visceral pain, intestinal barrier function, intestinal motility, and neurobehavior.^16^ Studies have shown that the gut not only participates in physiological activities such as digestion, metabolism, and immunity but also affects the organism in various ways.^17^ For example, it can influence behavior, cognition, mood regulation, pain perception, obesity, neoplasia, inflammation, and autoimmune diseases through the nervous system, endocrine system, immune system, and gut microorganisms (Figure 1).^18^, ^19^ It is worth noting that TBI can cause structural and functional damage to the gastrointestinal tract,^20^ and treatments that target the gastrointestinal tract can improve the neurological prognosis of TBI,^21^ which suggests that abnormalities in the gastrointestinal axis may be one of the causative mechanisms of TBI.

## 4 EFFECT OF TBI ON THE BGM AXIS

### 4.1 Microorganisms and metabolites

The human intestinal microbiota contains approximately 1014 bacterial species dominated by four phyla: Firmicutes, Bacteroidetes, Proteobacteria, and Actinobacteria, comprising greater than 95% of resident bacteria in healthy adults.^22^, ^23^ The human body and gut microbes have a mutually beneficial relationship.^24^ The intricate microbiota influences host health through maintenance of the gut, modulation of immune defense and inflammation, hormone secretion, and the involvement of metabolite products in the regulation of the BBB, myelin sheath formation, vagal excitation, and microglial cell maturation.^25^

It is reported that TBI significantly alters the gut microbiome, resulting in changes in the structural composition and biodiversity of the intestinal microorganisms,^26^, ^27^ decreasing commensal bacteria and increasing the presence of pathogenic bacteria, leading to “gut dysbiosis,” thereby promoting disease progression (Table 1).^28^, ^29^ This dysbiosis, in turn, directly and indirectly contributes to TBI‐induced outcomes, including loss of barrier integrity, chronic activation of local and systemic immune responses, and dysmotility.^30^ Preclinical studies have demonstrated that antibiotic‐induced depletion of gut microbiota before TBI can exacerbate outcomes, implying a protective effect of specific microbiota or microbial products in TBI.^31^ The benefits of manipulating the microbiota in TBI are supported since studies showed that pretreatment with *Lactobacillus acidophilus* or *Clostridium butyricum* ^32^ can attenuate TBI‐induced neurological effects, neuroinflammation, and neuropathology.^21^

**Table 1:** Effect of TBI on the microbiome.

References	Object	TBI method	Microbial sampling	Microbiome outcomes
Ma et al.^21^	C57BL/6J mice	Controlled cortical impact	Stool samples	TBI decreased *Lactobacillus gasseri*, *Ruminococcus flavefaciens*, and *Eubacterium ventriosum* microbial and increased *Eubacterium sulci*, *Marvinbryantia formatexigens*.
Nicholson et al.^26^	C57BL/6J mice	Repeated weight drop	Fecal pellets	α‐Diversity unchanged; time‐dependent change in β‐diversity; Decreased and increased relative abundance of phyla Firmicutes and Bacteroidetes.
Treangen et al.^27^	C57BL/6J mice	Controlled cortical impact	Fecal pellets	Genus‐level changes: Relative abundance of *Lactobacillus* decreased, *Marvinbryantia* and *Clostridia* increased; species‐level changes: relative abundance of *Lactobacillus gasseri*; *Ruminococcus flavefaciens*, and *Eubacterium ventriosum* decreased versus baseline levels; *Eubacterium sulci* and *Marvinbryantia formatexigens* increased. TBI reduced the relative abundance of *Lactobacillus gasseri* at versus sham and resulted in a loss of *L. gasseri*, *Lactobacillus johnsonii*.
Urban et al.^28^	Chronic TBI	TBI patients at two study sites	Stool samples	Shift in microbial community and increased α‐diversity in chronic TBI patients; phylum‐level changes: chronic TBI increased Actinobacteria, Firmicutes, and Verrucomicrobia and decreased Bacteriodetes; family‐level changes: chronic TBI increased Ruminococcaceae and Ruminococcaceae and decreased Prevotellaceae; species‐level changes: chronic TBI increased *Bacteroides thetaiotaomicron* and decreased *Prevotella copri*, *Prevotella* spp., and *Sutterella* spp.
Taraskina et al.^29^	Wistar outbred strain rats	Controlled cortical impact	Stool samples	The most pronounced decrease was observed in the Agathobacter species, the genera Faecalibacterium and Paraprevotella, and *Eubacterium coprostanoligenes*. The most significant post‐TBI growth in two Rikenellaceae gut‐group species, members of the Prevotellaceae family (including Prevotella), and *Lactobacillus*, *Turicibacter*, and *Helicobacter* species.

Abbreviation: TBI, traumatic brain injury.

Short‐chain fatty acids, one of the most abundant microbial metabolites in the intestinal lumen,^33^ are rapidly absorbed by the body and have an anti‐inflammatory effect on microglia, improving neurological recovery after ischemic brain damage.^34^ In addition, intestinal flora also produce neuroactive substances, such as γ‐aminobutyric acid and 5‐hydroxytryptamine, which serve as neurotransmitters and contribute to the recovery of neurological function after TBI.^35^ These findings highlight the complex relationship between gut microbiota and various disease outcomes, further emphasizing the idea that microbial populations can influence postinjury neuro‐pathophysiology and functional impairment through dysbiosis‐dependent mechanisms.

### 4.2 Initiation of the immune response

The intestine plays a vital role in the immune system of our body. The population of intestinal mucosal immune cells makes up approximately 70%–80% of the entire immune cell population of the organism.^36^

These immune cells interact in a complex way with microorganisms within the intestine. When TBI leads to an imbalance in the intestinal flora, immune cells trigger intestinal immune responses,^37^ releasing pro‐inflammatory cytokines that can reach the brain through the bloodstream or by other means, leading to neurological and endocrine system disruptions, possibly causing enteric neuropathy and other neuropsychiatric disorders.^8^, ^38^

After TBI, the most common symptoms include intestinal congestion and edema, reduced villous glands, disruption of the intestinal barrier, increased intestinal permeability, and the release of inflammatory factors such as interleukin‐1 (IL‐1), tumor necrosis factor‐alpha (TNF‐α), and chemokines from the injured intestinal tract.^39^ Inflammatory factors enter the BBB from the damaged intestinal barrier through blood and lymphatic circulation, leading to BBB disruption, capillary endothelial cell gap expansion, and activation of microglia and astrocytes.^40^ Activated microglia then stimulate the immune system to phagocytose cellular debris and necrotic tissues by releasing free radicals, proteases, and inflammatory chemokines, which promote tissue repair and remodeling.^41^

Microglia can be categorized into two types, M1 (pro‐inflammatory) and M2 (anti‐inflammatory). During the early stages of TBI, M2 microglia plays a dominant role; however, with prolonged activation, there is a gradual shift from M2 to M1 microglia,^42^ which release high concentrations of pro‐inflammatory factors, such as interferon‐gamma. Additionally, microglia release elevated levels of other pro‐inflammatory factors like TNF‐α, IL‐1, and chemokines, contributing to chronic neuroinflammation.^43^ This neuroinflammation further exacerbates oxidative stress and accelerates neurodegeneration, potentially weakening or worsening neurological recovery following brain injury.^44^ Furthermore, microglia and astrocytes phagocytose necrotic tissue, leading to scarring and subsequent irreparable damage to the brain tissue.^44^

Following a TBI, gut microbial dysbiosis can lead to enterogenic infections.^45^ This condition exacerbates brain injury by increasing microglia‐mediated inflammatory responses.^27^ In conclusion, the intestinal inflammatory response following a TBI, involving the activation of immune cells and the production of inflammatory mediators, among other factors, plays a role in the regulatory mechanism between the gut and the brain. By understanding these processes, we can enhance our understanding of disease onset and management.

### 4.3 Autonomic and ENS dysfunction

Perhaps the most direct pathway of communication within the BGM axis is among the gut microbiota, the ENS, and the vagus nerve. The brain and gut communicate directly through the vagus nerve and the autonomic nervous system in the spinal cord, as well as through the ENS, which establishes neural connections between the gut and the brain.^46^, ^47^

The autonomic nervous system consists of sympathetic and parasympathetic nerves, including the vagus nerve. Specific to TBI, disruption of the BGM axis can trigger subsequent complications. Among adult patients with TBI, 8%–33% of individuals exhibit autonomic imbalance.^48^, ^49^ TBI‐induced disruption of cortical connections, affecting the vagal complex, could result in dysautonomia, which seems to have a significant effect through the BGM axis. Similarly, TBI patients with dysautonomia sustain more complications and experience a worse prognosis.^8^ Disturbances of the intestinal vagal system in TBI patients may cause increased intestinal permeability, secondary to decreased expression of the intestinal tight‐junction proteins, such as zonulin‐1 and occludin.^50^ The resulting disruption of the anatomical and functional integrity of the gut can result in the development of systemic inflammation, bacterial translocation, and sepsis.^51^

The vagus nerve plays a dominant role in the autonomic nervous system and is also a vital component of the ENS.^52^ Approximately 90% of vagus nerves are located in the gut and extend to functional areas of the brain, forming the vagal cholinergic pathway, which plays a crucial role in controlling learning, memory, consciousness, behavior, and visceral function.^53^, ^54^ After TBI, dopaminergic neurons in the gastrointestinal tract become activated, causing the release of high levels of dopamine, with sympathetic neural excitation and vagal inhibition, ultimately affecting brain functions such as consciousness and movement.^4^

The intestinal vagus nerve can indirectly exert anti‐inflammatory effects by maintaining the intestinal barrier and balancing the inflammatory response. Research has shown that cervical vagotomy in mice can lead to the development or even exacerbation of intestinal inflammatory responses.^55^ On the other hand, vagus nerve stimulation (VNS) is known to have anti‐inflammatory effects by reducing serum endotoxin, IL‐1β, IL‐6, and IL‐8. This stimulation also helps in reducing intestinal permeability.^48^, ^56^ In addition, the vagus nerve can respond to harmful signals. Following a TBI, vagal afferents can act as a mechanism for inflammatory mediators, intestinal microbes, and their metabolites to affect the function of CNS through the BGM axis.^57^ Based on the combination of several domestic and international studies, it seems that stimulating the vagus nerve could be a potentially effective therapy for trauma. It is important to note that while several promising results have been found, relevant studies are still in their early stages of development, and the interaction with the BGM axis through which bioelectrical signals are mediated is still unclear.

### 4.4 Activated hypothalamic–pituitary–adrenal (HPA) axis

The HPA axis is the body's primary endocrine regulatory system and plays a crucial role in coping with trauma, stress, metabolism, and the immune system.^58^, ^59^ Activation of the HPA axis is triggered by posttraumatic stress, which has a significant impact on gut function. These changes cause alterations in microbial composition and diversity. On the other hand, research has shown that gut microbial dysbiosis can affect the function of the HPA axis by causing changes in glucocorticoid levels, as observed during in vivo equilibrium and the stress response in germ‐free mice.^60^, ^61^ Posttrauma tissue ischemia and oxygen deprivation can induce significant apoptosis and necrosis. This event can trigger the release of danger‐associated molecules and activate corresponding recognition receptors, such as Toll‐like receptors.^62^, ^63^ Inflammation may stimulate the production of pro‐inflammatory cytokines by microglial cells and macrophages. These cytokines can activate the neural conduction pathway, subsequently enhancing the activity of the HPA axis, and leading to the release of substantial quantities of catecholamines and glucocorticoid hormones. This, in turn, can impact intestinal permeability and microbial composition.^64^, ^65^

## 5 INSIGHTS INTO THE TREATMENT OF TBI IN THE CONTEXT OF THE BGM AXIS

Investigation into the BGM in the TBI has identified several promising targets for intervention. Considering the complexity of the bidirectional regulation of the BGM axis, we will only discuss the following possible treatment options to provide potential guidance for the treatment of TBI.

### 5.1 Microbial intervention (probiotics/prebiotics)

The reported link of gut microbiota to brain health and disorders raises the question of how to modulate the microbiota for re‐equilibration of the gut microbiota compositions altered by brain disorders. The structural composition and biodiversity of the gut flora undergo modifications following TBI,^26^, ^66^ which provides the theoretical justification for microbial intervention therapy. There is a growing body of evidence documenting the ability of probiotics and prebiotics to normalize the microbiota associated with neurological disorders.

Probiotics can regulate immune function, improve intestinal barrier function, generate organic acids and antimicrobial products, and interact with a host and its flora. It is reported that probiotic administration reduces BBB permeability and has neuroprotective effects in mice with TBI by upregulating tight junction proteins.^67^, ^68^ Probiotic supplementation has been demonstrated to improve behavioral and cognitive deficits in neurological disorders such as Alzheimer's disease and depression.^69^, ^70^ For the therapeutic effect of TBI patients, a few studies have found that oral probiotics can remove inflammatory mediators from the serum of TBI patients and improve anti‐inflammatory capacity, which can help to reduce the incidence of ventilator‐associated pneumonia and shorten the time spent in the intensive care unit.^71^ In addition, probiotics have also been found to reduce intestinal permeability by modulating the HPA axis and prevent lipopolysaccharide translocation by reducing stress response.^72^

Although great achievements were made, clinical translational trials still face challenges due to differences between rodent and human anatomy, diet, healthy microbiota, immunology, and social dynamics, among other factors. What is more, the efficacy of probiotics in TBI remains inconclusive, and the underlying mechanism is currently unclear. It may be that probiotics exert anti‐inflammatory effects by increasing IL‐10 production and decreasing the secretion of pro‐inflammatory cytokines by intestinal epithelial cells, which promotes glial cell proliferation.^73^, ^74^ All of these efforts provide theoretical support for microbiological interventions for the treatment of TBI, but they are still in their infancy, with more extensive research necessary to facilitate their clinical translation.

### 5.2 VNS

The vagus nerve, which is the primary parasympathetic branch of the autonomic nervous system and the tenth pair of cranial nerves, is essential for transmitting sensory information between various organs. It accumulates in the nucleus of the solitary tract of the brainstem and then connects with the dorsal vagal complex, hypothalamus, thalamus, hippocampus, amygdala, and medial prefrontal lobe. Studies have successfully treated refractory epilepsy and chronic treatment‐resistant depression with VNS.^75^, ^76^

The neuroinflammatory response following TBI is considered a key factor in secondary brain damage. Elevated levels of pro‐inflammatory cytokines, such as TNF, IL‐1, IL‐6, and IL‐8, are closely associated with secondary damage in acute TBI, which can result in BBB breakdown, brain edema, neuronal necrosis, and apoptosis.^77^ Several studies have demonstrated that VNS enhances cognitive and motor functioning, while also reducing secondary neuronal damage following TBI, revealing its neuroprotective effects.^78^

Aquaporins‐4 (AQP‐4) is an integral membrane protein expressed in glial cells, ventricular plasma cells, and capillary endothelial cells in the brain, and it is a critical regulator of water metabolism.^79^ Increased perivascular AQP‐4 expression during TBI leads to cerebral edema, which in turn increases intracranial pressure and impairs cerebrovascular perfusion.^80^ Due to this characteristic, several studies have been conducted to reduce cerebral edema in TBI treatment with the usage of VNS, which have yielded successful results.^81^, ^82^ Clough et al.^81^ in the TBI rat model of fluid percussion injury (FPI) found that FPI rats receiving VNS, though still showing moderate edema in the ipsilateral cerebral cortex at 2 days, had a marked and statistically significant attenuation of edema compared with the FPI no‐VNS rats. Besides, perivascular AQP‐4 expression was increased after TBI, which also indicated its role in brain edema.^83^, ^84^ Therefore, modulation of AQP‐4 may be another neuroprotective mechanism provided by VNS. In summary, the neuroprotective effect of VNS on TBI is established, but the treatment of behavioral and cognitive dysfunction post‐TBI is still in the preliminary stage. For VNS to be effective in treating TBI patients, future studies need to thoroughly investigate the interactions between the vagus nerve and the brain to maximize the efficacy of VNS in TBI patients.

### 5.3 Fecal microbiota transplantations (FMT)

As mentioned above, TBI can trigger intestinal microbiological disorders. The consequences of dysbiosis may be extensive, including impaired mucosal integrity, bacterial displacement, inflammatory states such as autoimmune responses, and changes in the function of the immune system both inside and outside the gut.^85^ Intestinal microdysbiosis also disrupts immune homeostasis by altering the balance of T cells, including Treg cells and γδT cells.^86^ Studies conducted on germ‐free mice have demonstrated that the “normal” gut microbiota contributes to intestinal motility, the integrity of the intestinal mucosa, and the differentiation of Treg cells.^87^ Therefore, approaches aimed at restoring the gut microbiota to its baseline microbial characteristics after TBI have become a reasonable target for treatment.

Transplanting healthy flora to the dysbiotic intestine through FMT is the most efficient way to restore the balance of intestinal flora and improve microbiota dysbiosis.^88^ FMT has proven to be successful in treating various intestinal disorders such as inflammatory bowel disease, irritable bowel syndrome, and Crohn's disease.^89^ Moreover, it has shown improvements in neurological functions related to Parkinson's and multiple sclerosis.^90^

In addition, FMT was found to have a neuroprotective effect on mice after stroke, possibly mediated by an increase in FoxP3+Treg cells in the gut and brain.^91^ This effect was not observed in a subset of lymphocyte‐deficient mice,^92^ supporting the idea that FMT works through immune regulation. On one hand, FMT represents a pioneering therapeutic approach to help ameliorate the dysbiosis and complications caused by TBI in patients, on the other hand, FMT is an experimental technique whose use for treating patients with TBI is still a matter of debate. FMT may disrupt the diversity of the baseline microbiota, leading to a collapse of the resistance to colonization by spectrally deleterious microorganisms, and may even increase the patient's risk of enteric‐origin infections. Therefore, when using this approach, it is necessary to carefully assess the patient's condition and conduct long‐term follow‐up to assess the effectiveness and safety of FMT in patients with TBI.

## 6 SUMMARY AND PROSPECTS

**Figure 2:**
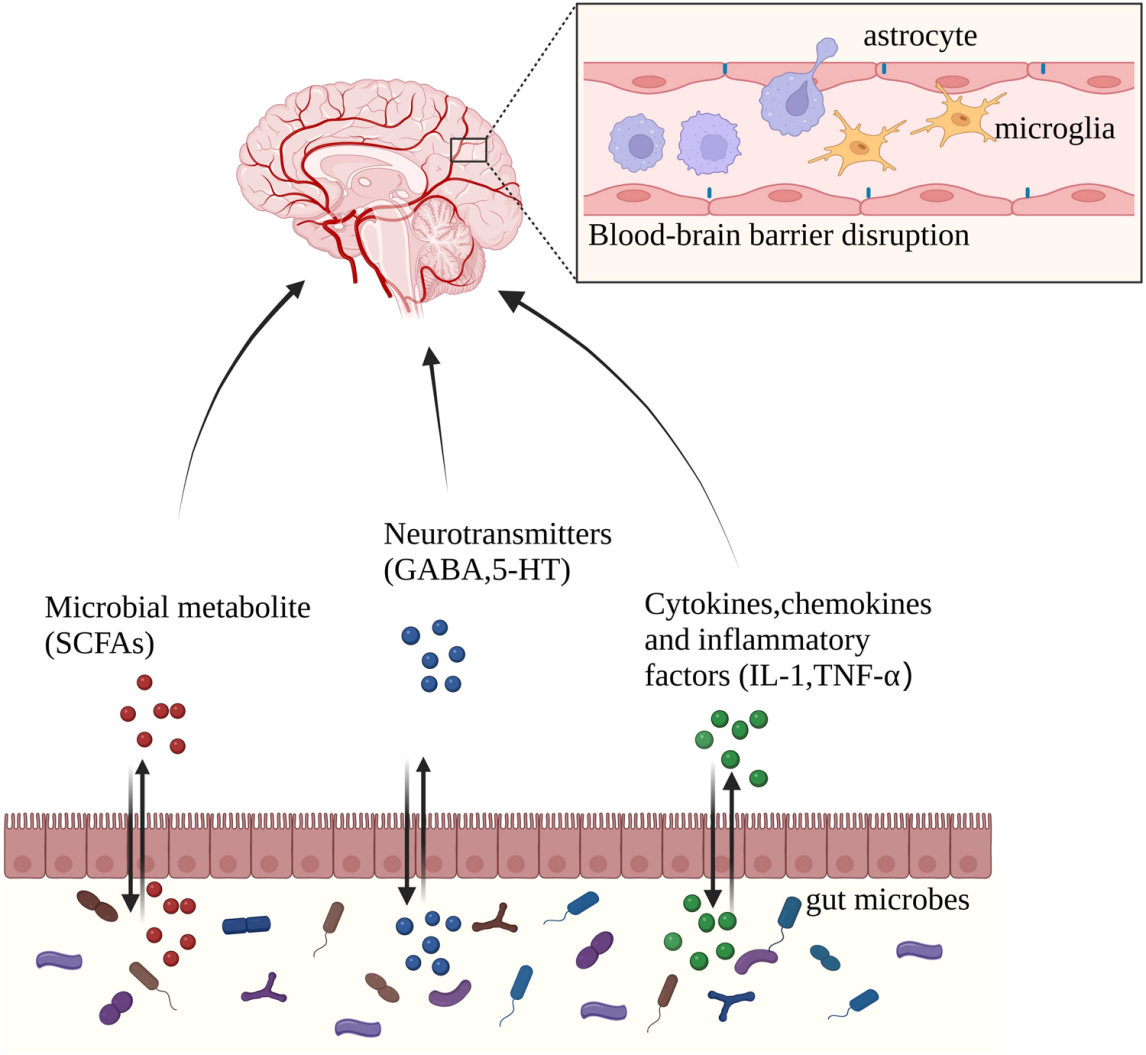
The mutual influence of the gut and brain after TBI. With the gut barrier damaged after TBI, gut microbes and metabolites reach the central system through the peripheral circulation, disrupting the blood–brain barrier and causing secondary damage through the induced inflammatory response. 5‐HT, 5‐hydroxytryptamine; GABA, aminobutyric acid; IL‐1, interleukin‐1; SCFAs, short‐chain fatty acids; TBI, traumatic brain injury; TNF‐α, tumor necrosis factor‐alpha. [Color figure can be viewed at wileyonlinelibrary.com]

TBI is a significant public health problem that can have devastating long‐term consequences for patients, including severe physical, cognitive, emotional, and/or behavioral disabilities. Through the disruption of the BGM axis and the intimate involvement of the gastrointestinal microbiota, TBI may initiate a feedback loop that potentiates a neuroinflammatory cascade and leads to secondary brain injury (Figure 2). Delightfully, the involvement of the BGM axis in the interaction between the brain and the gut after TBI represents a new field of research. Various methods have been developed to treat TBI, such as microbial intervention, flora transplantation, and vagal modulation, which show great therapeutic potential.

As we know, the adult nervous system is unable to recover from injury or regenerate to a large extent. Therefore, therapies based on the role of neural regeneration and repair have gained attention. Neural regeneration therapies are designed to promote the regeneration of damaged neural tissue through the use of neural stem cell therapies, growth factors, and cellular therapies, which can be combined with the modulation of the BGM axis to increase the efficiency of stem cell transplantation. In conclusion, neural regenerative therapies represent a prospective therapeutic area that could provide more effective treatment options for patients with TBI, but more research and clinical trials are needed to determine the optimal application and treatment strategy. Second, the concepts related to the BGM axis emphasize brain–gut interactions. Thus, another promising therapy after TBI could focus on delivering specific brain‐protecting nutrients to promote the repair and regeneration of damaged brain cells. However, limited research was reported on these two therapies. It is certain that the BGM axis provides a new direction for the treatment of TBI and promotes future research in new clinical intervention strategies. However, due to the relative complexity of TBI's pathophysiological mechanisms, there is still a long way to go before these novel therapies can be effectively translated into clinical practice.

## AUTHOR CONTRIBUTIONS

Jie Yu designed and wrote the manuscript. Yun‐Xin Chen and Jin‐Wei Wang prepared figures. Hai‐Tao Wu helped with proofreading and revision. All authors read and approved the final manuscript.

## CONFLICT OF INTEREST STATEMENT

The authors declare no conflict of interest.

## ETHICS STATEMENT

Not applicable.

## Data Availability

Not applicable as no new data are generated in this study.
